# The association between the size of adipocyte-derived extracellular vesicles and fasting serum triglyceride-glucose index as proxy measures of adipose tissue insulin resistance in a rat model of early-stage obesity

**DOI:** 10.3389/fnut.2024.1387521

**Published:** 2024-07-01

**Authors:** Jaime Delgadillo-Velázquez, Efrain Alday, María Magdalena Aguirre-García, Rafael Canett-Romero, Humberto Astiazaran-Garcia

**Affiliations:** ^1^Coordinación de Nutrición, Centro de Investigación en Alimentación y Desarrollo, Hermosillo, Mexico; ^2^Departmento de Ciencias Químico-Biológicas, Universidad de Sonora, Hermosillo, Mexico; ^3^Laboratorio de Inmunología Molecular y Cardiopatías, Facultad de Medicina, Instituto Nacional de Cardiología Ignacio Chávez, Unidad de Investigación UNAM-INC, Universidad Nacional Autónoma de México, Mexico City, Mexico; ^4^Departamento de Investigación y Posgrado en Alimentos, Departamento de Ciencias Químico-Biológicas, Universidad de Sonora, Hermosillo, Mexico

**Keywords:** early-stage obesity, insulin resistance, adipose tissue, high-fat diet, triglycerides, glucose, TyG index, adipocyte-derived extracellular vesicles

## Abstract

**Introduction:**

Obesity is a complex disease that predisposes individuals to cardiometabolic alterations. It leads to adipose tissue (AT) dysfunction, which triggers insulin resistance (IR). This suggests that people with obesity develop local IR first and systemic IR later. AT secretes extracellular vesicles, which may be physiopathologically associated with the development of IR. Our aim was to evaluate the effect of a high-fat diet on different parameters of adiposity in a rat model of early-stage obesity and to determine if these parameters are associated with markers of systemic IR. In addition, we sought to explore the relationship between fasting blood measures of IR (Triglycerides/High Density Lipoprotein-cholesterol [TAG/HDL-c] and Triglycerides-Glucose Index [TyG Index]) with the size of adipocyte-derived extracellular vesicles (adEV).

**Methods:**

We used a model of diet-induced obesity for ten weeks in Wistar rats exposed to a high-fat diet. Final weight gain was analyzed by Dual X-ray absorptiometry. Visceral obesity was measured as epididymal AT weight. IR was evaluated with fasting TyG Index & TAG/HDL-c, and adEV were isolated from mature adipocytes on ceiling culture.

**Results:**

In the high-fat diet group, glucose and triglyceride blood concentrations were higher in comparison to the control group (Log2FC, 0.5 and 1.5 times higher, respectively). The values for TyG Index and adEV size were different between the control animals and the high-fat diet group. Multiple linear regression analyses showed that adEV size can be significantly associated with the TyG Index value, when controlling for epididymal AT weight.

**Conclusion:**

Our results show that lipid and glucose metabolism, as well as the size and zeta potential of adEV are already altered in early-stage obesity and that adEV size can be significantly associated with liver and systemic IR, estimated by TyG Index.

## 1 Introduction

A recent study showed that the prevalence of obesity increased further post-pandemic, which is concerning (1). By 2030, it is estimated that 1 billion people worldwide will be obese (2). Obesity is excess body fat, which increases the risk of other chronic non-communicable diseases as a result (3). Although obesity is a multifactorial disease, it is ultimately caused by an imbalance between energy intake and expenditure. The carbohydrate-insulin model proposes that the hormonal response to a high-carbohydrate diet is a shift from energy processing to deposition, leaving fewer calories for metabolic needs (4, 5). Thus, excess calories are stored as triglycerides within adipose tissue (AT), either deposited in subcutaneous or visceral depots (6). Visceral fat has been associated with metabolic perturbations caused by metabolites produced in ectopic fat, such as proinflammatory cytokines, free fatty acids, and acylcarnitines (7–9). However, the mechanisms underlying the predominant increase of only one AT depot vs. the others have not been properly understood (8–10).

In the presence of obesity, the adipocyte reaches its maximum expansion capacity, resulting in the insulin resistance (IR) of AT. This leads to a failure of insulin to inhibit lipolysis, thereby increasing the release of free fatty acids, adipokines (leptin, adiponectin), and also the production of pro-inflammatory cytokines, such as interleukin (IL)-1 and IL-6, by adipose tissue-resident macrophages. These free fatty acids, adipokines, and cytokines interfere with insulin signaling, resulting in lipotoxicity and ectopic fat accumulation in tissues such as the liver and skeletal muscle (11, 12). The immuno-metabolic changes that occur in AT during IR contribute to metabolic dysfunction and exacerbate inflammation, thereby disrupting homeostasis (13, 14).

As an endocrine organ, AT also secretes adipocyte-derived extracellular vesicles (adEV), which play a crucial role in intercellular communication and metabolic regulation (15–17) and actively contribute to metabolic disorders by influencing signaling pathways and gene expression in target cells mediated either directly or through microRNAs (18–22). Research has shown that, in obesity, a phenotypic shift in AT macrophages from anti-inflammatory to pro-inflammatory states results in increased secretion of cytokines and microRNAs via adEV, exacerbating IR and affecting systemic glucose homeostasis (23–29). Furthermore, obesity could affect the biogenesis and secretion of adEV, altering their composition and potentially impacting recipient cell function, underlining the interconnected roles of adEV in cardiovascular disease and IR (30–32). Further investigations are necessary to explore the regulatory mechanisms of adEV on IR and to determine their potential as biomarkers and therapeutic targets in metabolic diseases.

The euglycemic-hyperinsulinemic clamp is considered the gold-standard test for insulin sensitivity assessment, as it measures peripheral glucose uptake during elevated insulin levels (33). However, it is time-consuming, expensive, and complex in nature, making it challenging to implement in larger population studies and clinical settings (34). Instead, fasting Triglyceride-Glucose Index (TyG Index) and the ratio of triglycerides/high-density lipoprotein-cholesterol (TAG/HDL-c) are used, as they are easy to carry out and are cost-effective (34, 35). These parameters reflect lipotoxicity and IR in the liver and other metabolically active tissues, which result from an increased fasting triglyceride and glucose secretion from this organ and impaired clearance by muscle and AT (35–38). Thus, we aimed to explore the relationship between the size of adEV and fasting blood measures of systemic IR (TAG/HDL-c and TyG Index) as potential biomarkers of IR in AT in a rat model in early-stage obesity.

## 2 Methods

### 2.1 Experimental model

Experiments were performed according to international standards for the proper care of laboratory animals (39) and approved by the Research Ethics Committee of the *Centro de Investigación en Alimentación y Desarrollo*, Hermosillo, Sonora (CEI/010–2/2022). Efforts were made to minimize the number of animals used for the study following the 3Rs principle (replacement, reduction, and refinement).

Sixteen 16-week-old male Wistar rats (300–350 g) were housed in groups of 4 per cage under controlled conditions (7 am/7 pm light–dark cycle, 22°C temperature, and 40–60% humidity). After 2 weeks of acclimatization, the rats were paired according to their body weight, and each rat was randomly assigned to a different group. Eight rats were assigned to one of the following groups: (1) standard diet, consisting of 4.36 kcal/g (17% kcal of dietary fat from porcine animal fat – LabDiet 5,008) for the control group (Ctrl group) and (2) a high-fat diet (HFD), consisting of 4.73 kcal/g (45% kcal of dietary fat from lard and soybean oil – Research Diets D12451) for the intervention group (HFD group). The experiment lasted 10 weeks. Food and water were available *ad libitum*.

Body weight was recorded throughout the experiment, and weight gain was calculated weekly. Food consumption was recorded every week, and the data were used to calculate the energy intake. In addition, the food efficiency (FE) and the food consumption efficiency rate (FER) were also calculated weekly according to the equations:

(1) FE = weight gain (g) / caloric intake (kcal).
(2) FER = weight gain (g) / feed intake (g).

Body mass index (BMI g/ cm^2^) and Lee index (g/mm) were calculated at the beginning and at the end of the intervention, using body weight and length (snout-anus) data.

At the end of the experiment, rats were fasted for 12 h and anesthetized with an intraperitoneal injection of ketamine (40 mg/kg) and xylazine (3 mg/kg) to perform body composition analysis by Dual X-ray absorptiometry (DXA).

### 2.2 Dual X-ray absorptiometry

The sedated experimental units were placed in the DXA equipment (Discovery QDR Series, Hologic Discovery 87,899, Danbury, United States) previously calibrated with a Hologic Rat Step Phantom (P/N 010–0758). Fat mass was expressed as percentage (%) and total content (g) relative to total body weight. Total lean mass without bone was determined by subtracting the mineral content from the fat-free mass (40).

### 2.3 Biochemical parameters and tissue collection

After completion of the body composition study, the animals were returned to their individual cages, with food and water supplied *ad libitum*. Animals were fasted at the beginning of the light cycle and sedated for euthanasia 6 h later (1 pm). Using a biosafety cabinet, a blood sample was drawn into a vacutainer tube and the serum was stored at −80°C for later determination of glucose, triglycerides (TAG), total cholesterol, high-density lipoprotein cholesterol (HDL-c), and low-density lipoprotein cholesterol using commercial kits following manufacturer’s instructions (BioSystems, Barcelona, Spain). After confirming the cardio-respiratory failure of each animal, the liver and epididymal adipose tissue (EpAT) were extracted (41). The tissues were weighed on a calibrated digital scale and stored at −150°C.

### 2.4 Metabolic characterization

Abdominal obesity was assessed using the ratio of EpAT weight to total body weight (BW). IR was evaluated using the TAG/HDL-c ratio and the TyG Index, which was computed according to the following equation (34):

(3) TyG Index = ln [TAG (mg/dL) *Glucose (mg/dL)]/2.

### 2.5 Adipocyte-derived extracellular vesicles isolation and characterization

To obtain mature adipocytes, we initially performed AT digestion using the methodologies developed by Rodbell (42) and Jumabay et al. (43), with some adaptations. First, 5 g of EpAT was weighed, minced and hydrated with Hanks’ Balanced Salts Solution (biowest, X0513), supplemented with +0.35 g/L NaHCO_3_ + 20 g BSA (Sigma, Fraction V BSA) conforming the “Washing Buffer HBSS.” Next, EpAT digestion was performed with the same buffer solution but with added Collagenase I Worthington CLS-1 (1 mg/mL) to reach a buffer volume of 3x the EpAT (5 g EpAT/15 mL Digestion buffer at 37°C), which was then agitated for 1 h while incubated at 37° C, 5% CO_2_. Halfway through digestion, the tube was briefly removed from the incubator, gently shaken manually for 30 s, and returned to the incubator to complete the full hour. Subsequently, the homogenate was filtered through a medical mesh to remove clumps, washed with 5 mL of washing buffer, and the effluent was collected in falcon tubes and centrifuged at 200 x g for 7 min. The mature adipocytes floated to the top and were drained from the bottom with an 18G syringe. They were then rinsed to reach a volume of 15 mL, and the centrifugation was repeated for 3 more washes. Lastly, the mature adipocytes were drained and placed with a Pasteur pipette into cryo-vials to be stored at −80° C. All work was carried out using a laminar flow hood at all times.

The ceiling culture technique was used to isolate EVs from mature adipocytes (44). Dulbecco’s Modified Eagle’s Medium + Fetal Bovine Serum Exo-free (System Biosciences, EXOFBSHI-250A-1) 10% v/v culture medium was prepared under sterile conditions. In a 6-well plate, 2 mL were placed in each well at 37° C, and 150 uL of mature adipocytes and a coverslip were placed over each well. The plate was kept in an incubator at 37° C, 5% CO_2_, for 72 h. Following the precipitation method, the supernatant was removed to isolate adEV, using the ExoQuick-TC kit (EQULTRA-20TC-1) following the manufacturer’s instructions.

According to international guidelines for the study of extracellular vesicles (45), the characterization consisted of the following: (1) analysis of the size and zeta potential using dynamic light scattering (DLS) with the software DYNAMICS 7.3.1.15 (Möbiuζ, Wyatt Technology Corp., Santa Barbara, CA, United States); (2) analysis of specific proteins of extracellular vesicles using the commercial ExoCheck kit (EXORAY210B-8) following manufacturer’s instructions; and (3) morphological evaluation by negative stain transmission electron microscopy using the JEOL JEM-1011 microscope (Jeol, Ltd., Tokyo, Japan). For the above #2 & #3 analysis, two adEV samples per group were randomly used (Supplementary Figures S1, S2).

### 2.6 Statistical analysis

Results were expressed as mean ± standard error of the mean (SEM) or median and inter-quartile range (IQR). GraphPad Prism version 8 (GraphPad Software, San Diego, CA, United States). Group comparisons were made using the Student’s t-test, the Mann–Whitney test, and/or the Wilcoxon test (for values from the same group), depending on the distribution of the data. A *p* < 0.05 was regarded statistically significant.

Multiple linear regression analyses were used to determine the association between the variables of interest, adjusting for possible confounders. The dependent variables used were TyG Index and TAG/HDL-c. The independent variables of interest were the size and Zeta potential of adEV. The confounders considered due to their possible influence on IR were weight gain, amount of visceral adipose tissue, body fat, and type of diet. The regression modeling was performed in STATA (v15.0 StataCorp LP, College Station, TX, United States), filtering variables by constructing linear regression models between each dependent and independent variable. The variables with biological plausibility and whose beta coefficients in simple regression had a *p*-value <0.2 were used to construct the multiple linear regression models using a stepwise approach with a p-value of entrance ≤0.05 and a p-value of rejection >0.05. In case confounders were found significant, collinearity and interaction were tested. Assumptions of the model were assessed through graphic examination of residuals. Additional models (S1-S4) were generated by forcing the entrance of rejected variables to assess the sensitivity of the model to their inclusion. The Akaike and Bayesian criteria were used to choose the best models.

For EpAT/BW, TyG Index, and adEV size, the values that best distinguished between the Ctrl and HFD groups were obtained for the index with the higher area under the receiver-operating characteristic curve (AUC), based on Youden’s statistic. The above analyses were performed in R Core Team (2023).

## 3 Results

### 3.1 High-fat diet promotes higher weight gain with lower food and caloric intake

**Figure 1:**
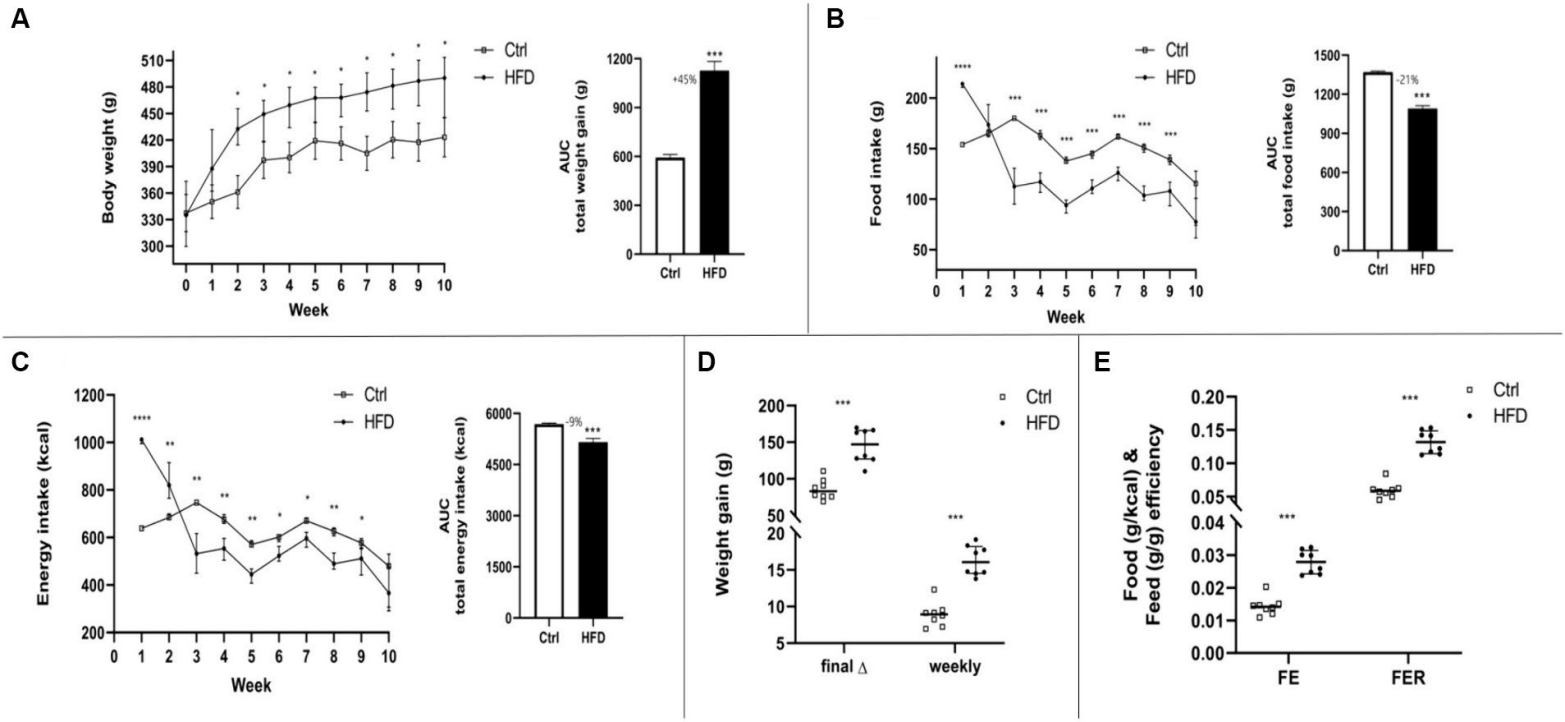
Dietary effect on body weight **(A)**, food intake **(B)**, and energy intake **(C)** in rats, comparing a control (Ctrl) vs. high-fat diet (HFD) over a 10-week period, and their respective areas under the curve (AUC). Final and average weekly weight gain **(D)**, food efficiency (FE), and food consumption efficiency rate (FER) **(E)**. HFD (*n* = 8) and Ctrl (*n* = 8). Data are presented as mean ± SEM; Student’s *t*-test HFD vs. Ctrl. * *p* < 0.05, ** *p* < 0.01, *** *p* < 0.001, ****p < 0.0001.

When compared to the Ctrl group, the HFD group showed a significant increase in body weight from week 2 until the end of the 10-week intervention (Figure 1A). However, food consumption and caloric intake were higher in the Ctrl group (Figures 1B,C). Despite this, the average weekly weight gain or the cumulative weight gain at the end of the study was significantly higher in the HFD group (Figure 1D). It is noteworthy that the HFD group showed a significantly higher FE and FER (Figure 1E).

### 3.2 Effect of weight gain on body composition and EpAT

Table 1 shows that the BMI and Lee index in both the Ctrl and HFD groups were higher at the end of the 10-week study. However, at the end of the intervention, the BMI in the HFD group was significantly greater than in the Ctrl group. Fat and lean masses were also significantly larger in the HFD group than in the Ctrl group. Similarly, EpAT was bigger in the HFD group compared to the Ctrl group. With these results we confirm that the model did show obesity. Following the Youden index analysis, the percentage value of EpAT that distinguished control from HFD-fed animals was >0.02% of total body weight, providing the best trade-off (AUC = 0.98) between sensitivity (0.875) and specificity (0.875) to identify abdominal obesity.

**Table 1:** Changes in fat mass, lean mass, and relative tissue weight in the Ctrl and HFD groups at the start and at the end of a 10-week intervention.

	Ctrl (*n* = 8)	HFD (*n* = 8)
BMI (g/cm^2^)		
Initial	0.53 (0.50–0.55) ++	0.51 (0.46–0.54) ++
Final	0.65 (0.63–0.69)	0.70 (0.68–0.73) *
Lee Index (g/mm)		
Initial	2.75 (2.71–2.81) ++	2.70 (2.57–2.77) ++
Final	2.95 (2.93–3.02)	2.99 (2.93–3.06)
DXA		
Total body fat (%)	13.40 (7.92–16.85)	16.75 (15.33–19.40)
Total fat mass (g)	59.60 (31.83–77.35)	85.40 (75.48–92.40)*
Total lean mass (g)	369.0 (339.5–378.2)	389.2 (376.6–413.4)*
Tissue weight/body weight (BW)		
Epididymal Adipose weight/BW	0.016 (0.012–0.017)	0.022 (0.021–0.025)***
Liver weight/BW	0.027 (0.025–0.030)	0.027 (0.025–0.028)

Ctrl, Control; HFD, High Fat Diet; BW, Body weight; DXA, Dual X-ray absorptiometry; presented values correspond to the median (IQR), *n* = 16; Wilcoxon test initial vs. final in the same diet group ++p < 0.01; Mann–Whitney test HFD vs Ctrl *p < 0.05; ***p < 0.001.

### 3.3 Dietary effects on fasting serum glucose concentrations and lipid profile and their relationship with IR in the HFD group

**Figure 2:**
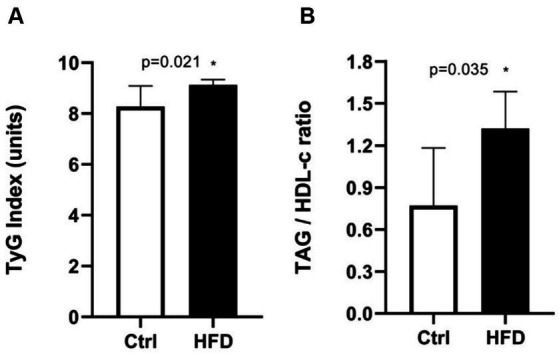
Insulin resistance through TyG Index **(A)**, TAG/HDL-c ratio **(B)**. Experimental groups: Control (Ctrl) and High-fat diet (HFD). Each bar represents the median (IQR), (*n* = 8 per group). Mann–Whitney test, *p < 0.05.

We analyzed the development of IR in the Ctrl and HFD groups. As Figure 2 shows, the TyG Index was significantly greater in the HFD group [9.1 units (IQR 8.9–9.3)] compared to the Ctrl group [8.3 units (IQR 7.7–9.0)]. This result was also repeated in the TAG/HDL-c ratio [Ctrl 1.32 (IQR 1.04–1.58) vs. 0.77 (IQR 0.57–1.18)]. However, no differences in total cholesterol, low-density lipoprotein cholesterol, and HDL-c concentrations were found between groups (data not shown). For this reason, we assessed IR using only the TyG Index and following the Youden index analysis. The value of the TyG Index that distinguished HFD-fed animals from Ctrl was >8.55 units, providing the best trade-off (AUC = 0.84) between sensitivity (1.00) and specificity (0.63) for identifying IR.

### 3.4 Size of extracellular vesicles related to the degree of IR

**Figure 3:**
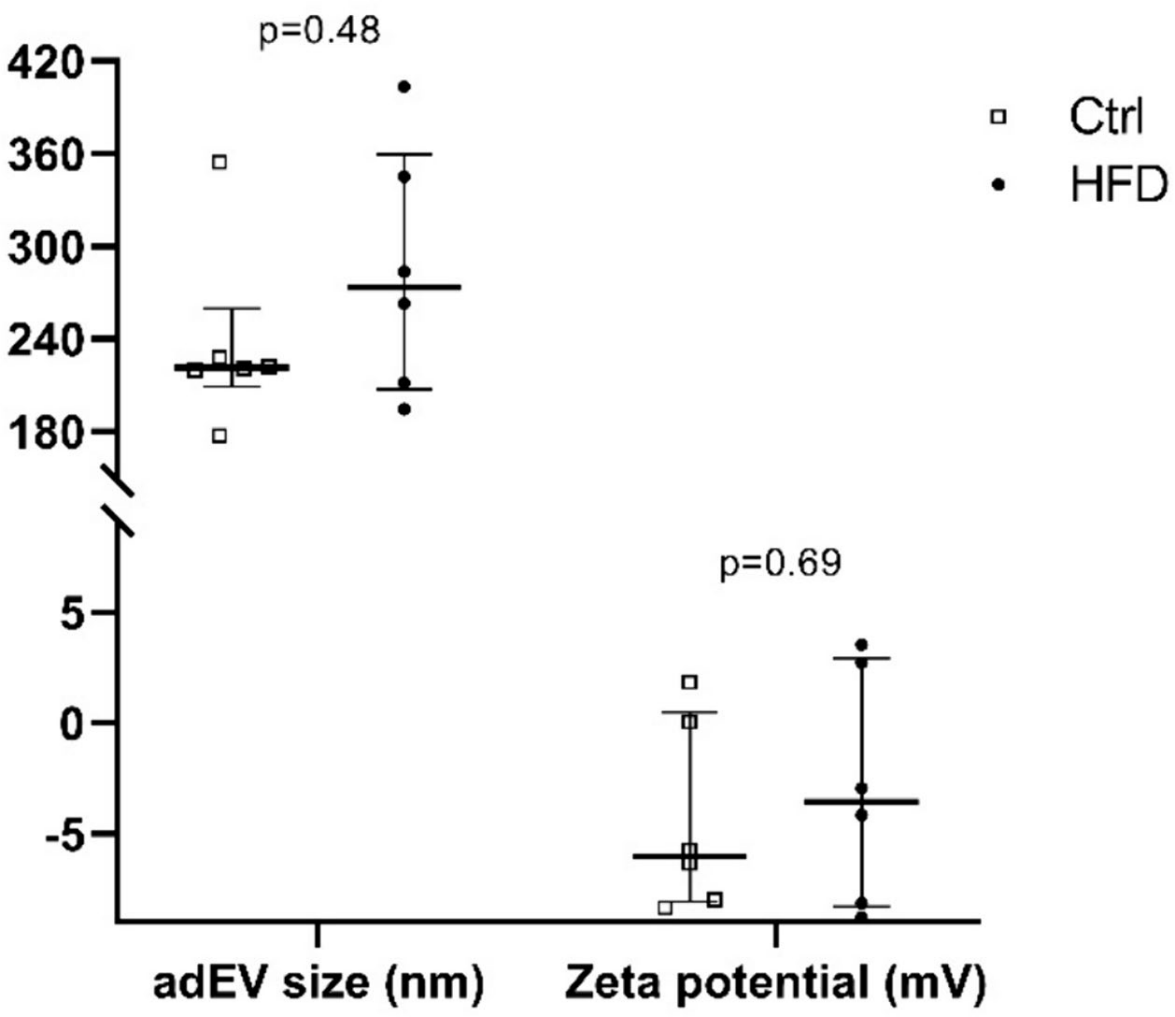
Size and zeta potential of adipocyte-derived extracellular vesicles (adEV) between groups. Ctrl: Control (*n* = 6); HFD: High Fat Diet (*n* = 6). Values correspond to the median (IQR), *n* = 12; Mann–Whitney test; HFD vs. Ctrl.

Figure 3 shows the size as well as the zeta potential of adEV found in the Ctrl and HFD groups. Though not significant (*p* > 0.05), the HFD group showed a larger median size [273.2 nm (IQR 207.2–359.7) vs. 221.3 nm (IQR 209.0–259.5)] and lower negative zeta potential [−3.56 mV (IQR -8.30 – +2.93) vs. -6.03 mV (IQR -8.35 – +0.49)] of adEV compared to the Ctrl group. In addition, following the Youden index analysis, the value of the size of adEV that distinguished HFD-fed from control animals, providing the best trade-off (AUC = 0.64) between sensitivity (0.67) and specificity (0.83), was >262.86 nm.

**Figure 4:**
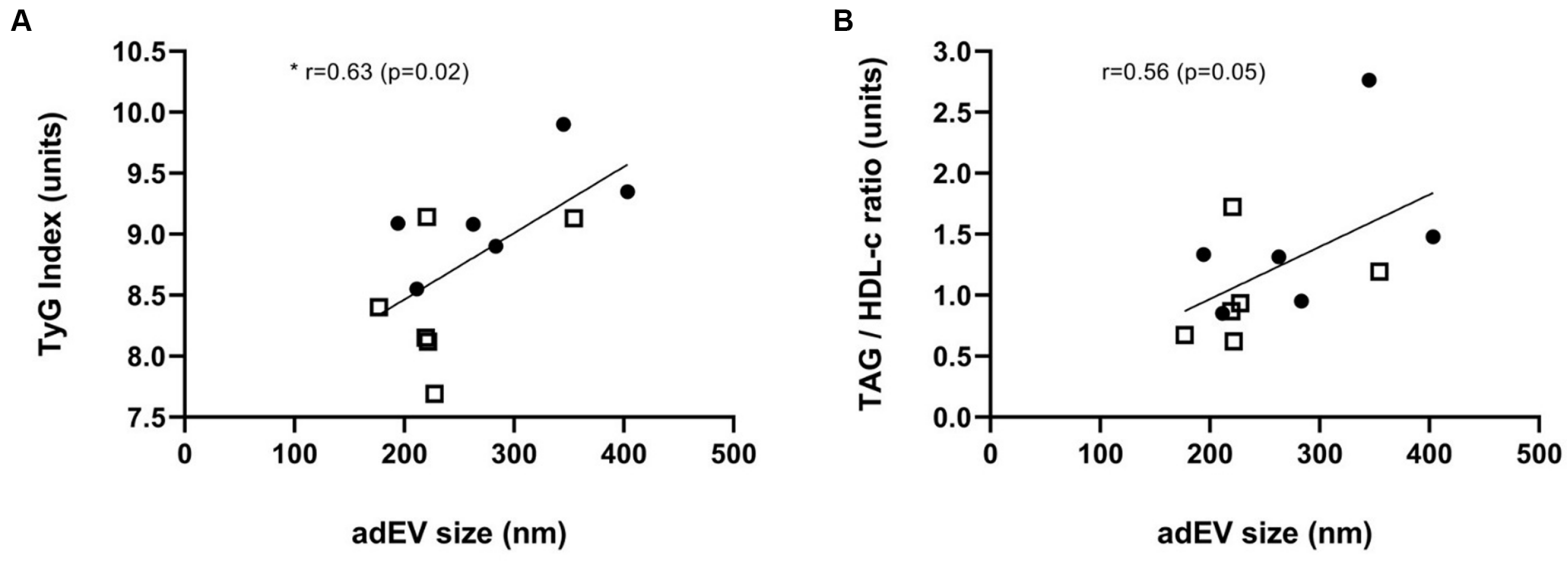
Insulin Resistance correlates with the size of adipocyte-derived extracellular vesicles (adEV). Relation between TyG Index **(A)** & TAG/HDL-c **(B)** with size of adEV; r, correlation coefficient; *p value <0.05; White square (Ctrl group), Black filled circles (HFD group); *n* = 12.

Furthermore, we analyzed the size of adEV and its association with markers of IR by TyG Index and TAG/HDL-c. A correlation analysis between the TyG Index and vesicle size showed a positive and significant relationship (Figure 4A). A borderline significant correlation was found in the case of the TAG/HDL-c marker (Figure 4B).

### 3.5 The relationship between parameters of adiposity and markers of IR

We explored the univariate association between TyG Index and TAG/HDL-c with independent variables of adiposity (Table 2). A logarithmic transformation (log10) to normalize data was used for epididimal fat relative to body weight (EpAT/BW) and TAG/HDL-c. Correlation analysis showed that the size of adEV was significantly and positively correlated with IR, but not the zeta potential. Relating to the other related variables, all were significantly associated with IR, except for body fat (%) in relation to TAG/HDL-c log_10_.

**Table 2:** Correlation and univariate regression analysis according to TyG Index and TAG/HDL-c.

	TyG Index			TAG/HDL-c log_10_		
Variable (X independent)	Correlation R coefficient	Regression Β coefficient	*p* value	Correlation R coefficient	Regression Β coefficient	*p* value
adEV size (nm)	0.63	0.0054448	0.029*	0.56	0.001422	0.059
zeta potential (mV)	0.36	0.0483002	0.251	0.28	0.0111696	0.372
Final weight gain (g)	0.61	0.012585	0.012*	0.59	0.0035787	0.016*
Body weight (g)	0.71	0.0132208	0.002*	0.59	0.0032695	0.014*
EpAT/BW log_10_	0.74	3.897858	0.001*	0.59	0.9188731	0.015*
Body Fat (%)	0.56	0.0891734	0.023*	0.36	0.0166882	0.172
FE (kcal/g)	0.58	69.41729	0.018*	0.57	19.95299	0.021*
Group (HFD)	–	0.8564183	0.014*	–	0.2117414	0.047*

adEV, adipocyte-derived extracellular vesicles; EpAT/BW, epididymal adipose tissue/body weight; FE, Food efficiency rate; HFD, High- fat diet; TyG Index, Triglyceride-Glucose Index; TAG/HDL-c: Triglyceride/High-density lipoprotein cholesterol ratio; *p < 0.05.

Following construction of the univariate regression models, and because zeta potential and food intake were not associated with the outcome variables (Table 2), we tested the association of the remaining explanatory variable (adEV size) with both outcome variables (TyG Index and TAG/HDL-c log10) of IR, adjusting for possible confounders. To do this, we constructed various multivariate linear regression models, which are shown in Table 3 (for TyG Index) and Supplementary Table S1 (for TAG/HDL-c).

**Table 3:** Multivariate regression analysis assessing associations with TyG Index.

	Model SW		Model S1		Model S2		Model S3		Model S4	
TyG Index (units)	Β	*p* value	Β	*p* value	Β	*p* value	Β	*p* value	Β	*p* value
adEV size (nm)	0.0049	0.008	0.0038	0.088	0.0045	0.054	0.0051	0.039	0.0052	0.014
Final weight gain (g)	–	–	0.0084	0.076	–	–	–	–	–	–
Body weight (g)	–	–	–	–	0.0068	0.117	–	–	–	–
EpAT/BW log_10_	3.774	0.006	–	–	–	–	–	–	4.276	0.036
Body Fat (%)	–	–	–	–	–	–	0.0398	0.293	–	–
Group (HFD)	–	–	–	–	–	–	–	–	−0.132	0.706

adEV, adipocyte-derived extracellular vesicles; EpAT/BW, epididymal adipose tissue/body weight; FE, Food efficiency rate; HFD, High-fat diet; TyG Index, Triglyceride-Glucose Index. Model SW was obtained with a stepwise approach. Models S1 to S4 tested the sensitivity of model SW by adjusting it for each of the variables rejected by the stepwise approach. Values correspond to the beta coefficient.

In Model SW (Table 3), the TyG index was significantly associated with adEV size, adjusting for EpAT/BW log_10_. Models S1, S2 and S3 refer to adEV size, adjusted by one covariate each (final weight, body weight, and body fat, respectively). In the last model (S4), TyG Index was significantly associated with adEV size, adjusted by 2 covariates: visceral fat and type of diet. Adjusting for weight gain and body weight, the adEV size almost reached a statistically significant difference (Models S1 y S2) to predict TyG Index. In contrast, the TAG/HDL-c marker was not associated with any independent variable of interest (Supplementary Table S1).

Results from the multivariate linear regression models showed that the best model was the one with adEV size and EpAT/BW as independent variables that explain the dependent variable TyG Index as a marker of systemic IR (Model SW), according to the value of Bayesian and Akaike criteria and the lowest *p* value.

## 4 Discussion

In this study, using a model of diet-induced obesity in Wistar rats, adEV size was significantly associated with the TyG Index, adjusted by the amount of visceral adiposity (EpAT/BW). We also found that the type of diet slightly reduced the significance of adEV size and EpAT/BW in the association model with TyG Index. Even though there was no significant difference in adEV size between groups, the multiple regression results suggest that the type of diet may mediate the effect of adEV size in leading to the development of systemic and liver IR. Additionally, the amount of body fat reduced the significance of the association between adEV size and TyG Index, indicating that the effect of adEV size could be partly independent of the total increase in fat mass.

It is well known that AT secretes EVs. These nanoparticles are recognized for their role in intercellular communication, transporting various biomolecules (15). Also, they serve as vehicles for releasing degradable substances (16, 17). Research has shown that adEV are not merely a consequence, but rather a causal factor in metabolic disorders. This is attributed to their molecular composition, which has the capacity to modulate signaling pathways and gene expression in target cells or tissues (18, 19), either directly or indirectly through microRNAs originating from AT (20, 21). In cases of obesity, there is frequently a transition in AT macrophages from an anti-inflammatory M2 phenotype to a pro-inflammatory M1 phenotype (20). This shift results in heightened secretion of biomolecules encapsulated within adEV. Furthermore, dysfunction of AT linked to obesity alters the size of adipocytes and molecular content of adEV, influencing recipient cells or tissues as a result (30, 31). These factors have the potential to induce local IR, thereby impacting systemic glucose homeostasis (12, 19, 23–25).

Kranendonk et al. (26) found that adEV release pro-inflammatory cytokines, affecting Akt phosphorylation in the liver and potentially contributing to IR. Meanwhile, Eguchi et al. (27) demonstrated that adEV from obese individuals may activate M1 macrophages and contain Perilipin A, suggesting this molecule as a potential biomarker for adEV. Since adEV could potentially be involved with proteins and genetic materials that could participate in cytokine production or insulin signaling pathway, especially in terms of glucose and lipid regulation (30, 46), they have been implicated in the development of cardiovascular disease (28, 29). Hence, it appears that adEV are essential for both local and distal intercommunication in the development and regulation of IR associated with obesity (30, 31).

There is limited evidence that associates characteristics such as content, concentration and size of EVs with metabolic biomarkers such as blood glucose, triglyceride concentrations, and oral glucose tolerance test, etc. In this sense, a study in individuals with obesity demonstrated that there is a positive correlation between the level of circulating EVs and IR (measured by the Homeostatic Model Assessment for insulin resistance; HOMA-IR), and also, with other metabolic parameters, such as blood glucose, triglyceride concentrations, and oral glucose tolerance test (32). On the other hand, Kwan et al. (31), has been reported that obesity influences the biogenesis and secretion of adEV, thereby modifying the intracellular content of non-esterified fatty acids and ceramides. These molecules play crucial roles in the assembly mechanisms of EVs, potentially altering their biophysical properties such as size, zeta potential, and concentration (31). The above agrees with our results.

In our study, the HFD group exhibited a larger size of adEV compared to the Ctrl group, but the size difference was not statistically significant, most likely due to the small sample size. This finding aligns with previous research by Blandin et al. (47), who observed increased adEV size in a murine model of diet-induced obesity compared to those on a standard diet, and even larger sizes compared to a genetic model of obesity, alongside higher adEV concentration per experimental unit (47). This highlights the significant influence of diet on both the number and size of adEV, potentially implicating them in the pathogenesis of obesity and its associated conditions.

While we did not confirm a higher adEV count due to methodological differences, specifically having used the dynamic light scattering instead of nanoparticle tracking analysis, we did observe a lower negative zeta potential in the HFD group compared to the Ctrl group, but again this result was not statistically significant. Zeta potential, indicative of EVs surface charge, could affect their interactions with other vesicles or target cells, influencing content internalization (31, 48). Notwithstanding this, zeta potential remains underreported in studies of EVs, and their alterations may depend on the cell of origin and membrane lipid composition (49, 50). While our study did not reveal significant differences in the zeta potential and size of adEV between the two groups, again, most likely due to the small number of experimental units, a difference was still observed, and should be corroborated in a future study. Considering that changes in adEV size and zeta potential could be significant indicators for monitoring and diagnosing cardiometabolic diseases (51, 52), further research is necessary to confirm whether alterations in the biophysical characteristics of adEV correlate with functional outcomes in the regulatory mechanisms of IR. This requires employing a standardized methodology for comprehensive analysis.

In IR, the interplay of macronutrient consumption plays a significant role. Chronic intake of fats and carbohydrates, perpetually activating anabolic pathways and contributing to the IR phenotype, may indicate metabolic inflexibility (53). This inflexibility could extend to other dietary amino acid-derived molecules, potentially deregulating the PI3K/Akt/mTOR signaling pathway (54). Consequently, the organism may initiate catabolic pathways to manage an excess of TAG, leading to elevated circulating glucose and TAG concentrations (55). Consistent with prior studies, our findings revealed a nearly twofold increase in the TAG/HDL-c ratio in the HFD group compared to the Ctrl (56, 57). However, individual lipid profile differences were not observed, as reported by Moreno-Fernandez (58). Nonetheless, without assessing insulin concentrations or conducting glucose tolerance tests, these conclusions remain unconfirmed. Further investigations are necessary to determine if disruptions in glucose and TAG metabolism originate in AT before impacting the liver and skeletal muscle, crucially validating the occurrence of mechanisms stemming from AT-IR in early-stage obesity.

Finally, plasma concentrations of glucose and TAG are commonly evaluated in clinical laboratories, with various suggested cut-off values for the TyG Index depending on individual sex and age (59, 60). Recent research has highlighted the TyG Index’s efficacy in predicting and evaluating prediabetes, surpassing insulin-dependent indices like HOMA-IR, and it serves as a valuable screening tool for investigating IR (36, 37, 61–63). Our study also found a correlation between the TyG Index and body fat, which is associated with visceral AT. Since DXA does not differentiate between body fat depots, these findings may aid in monitoring and assessing abdominal obesity in animal models. Also, values of the TyG index and size of adEV that distinguishes the Ctrl group from HFD could be used in future research.

## 5 Strengths and Limitations

The strengths of the present study are many and include: the use of non-isocaloric but commercial diets, a matched randomized control group from the beginning, reducing possible biases, and the analysis of body composition by DXA. Furthermore, standardized procedures for adEV isolation and characterization in the present study provide a fingerprint for specific proteins, such as annexin V, flotillin 1, TSG101, and ICAM, all suitable for the standardization of the study of adEV (Supplementary Figure S1).

By the same token, we acknowledge that we used a very small sample size (6 units per group), which can limit the statistical power to detect significant relationships and can also affect the stability and generalizability of regression model estimates. Also, the use of a single rat strain, age, and sex do not allow for the generalization of our results. Thus, the results should be taken in this context and would be more directly applicable to animal populations with similar characteristics. Another limitation is the type of diet used. Our diet had 45% energy intake from fat, which does not reflect the regular cafeteria fat consumption. Thus, a direct extrapolation of the results to humans is not appropriate.

## 6 Conclusion

Both in univariate and multivariate models, adEV size significantly predicted TyG Index. However, this was not shown for zeta potential or TAG/HDL-C ratio (most likely due to the small sample size). There was a borderline significance between adEV size and TAG/HDL-C ratio in the univariate model, but when the other measures of general or visceral adiposity were included, the relation did not reach significance. A larger sample size is needed to confirm our findings.

Our results highlight that there is a significant association between the size of adEV and the TyG Index. This last variable represents an economical and straightforward surrogate marker for both hepatic and systemic IR. These findings imply that size of adEV may be implicated in the early AT changes with HFD in a rat model and they may influence the development of systemic IR. Given the potential of these insights, the adEV could be instrumental for the development of a screening test for IR associated with AT dysfunction in early-stage obesity, enhancing early detection and intervention strategies.

## Data availability statement

The original contributions presented in the study are included in the article/Supplementary material, further inquiries can be directed to the corresponding author/s.

## Ethics statement

The animal study was approved by Comité de Ética en Investigación, Centro de Investigación en Alimentación y Desarrollo, Campus Hermosillo, Sonora, México. The study was conducted in accordance with the local legislation and institutional requirements.

## Author contributions

JD-V: Conceptualization, Writing – original draft, Writing – review & editing. EA: Resources, Writing – review & editing. MA-G: Visualization, Writing – review & editing. RC-R: Writing – review & editing. HA-G: Conceptualization, Project administration, Writing – review & editing.
