# Supplementary material for: The association between the size of adipocyte-derived extracellular vesicles and fasting serum triglyceride-glucose index as proxy measures of adipose tissue insulin resistance in a rat model of early-stage obesity
Source: Front Nutr. 2024 Jul 1;11:1387521. doi: 10.3389/fnut.2024.1387521 (PMC11247012; doi:10.3389/fnut.2024.1387521)
Supplement: Supplementary file 1 [file Image_1.pdf]

Supplementary materials

FIGURE 1S.

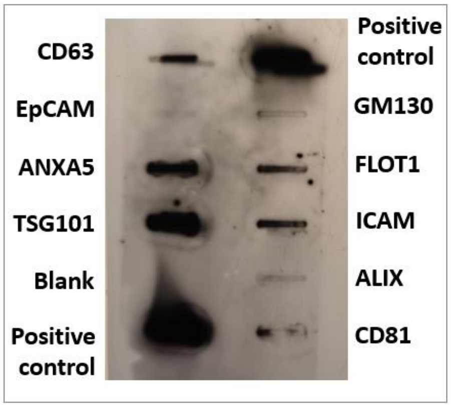

E**xo-Check ™ Array; 12-preprinted wells**:
8 antibodies for known exosome markers and
4 control wells (2 HRP positive, GM130 and blank).
50 ug of protein re-suspended in 1x PBS and lysed.
