# Supplementary material for: The association between the size of adipocyte-derived extracellular vesicles and fasting serum triglyceride-glucose index as proxy measures of adipose tissue insulin resistance in a rat model of early-stage obesity
Source: Front Nutr. 2024 Jul 1;11:1387521. doi: 10.3389/fnut.2024.1387521 (PMC11247012; doi:10.3389/fnut.2024.1387521)
Supplement: Supplementary file 2 [file Image_2.pdf]

FIGURE 2S

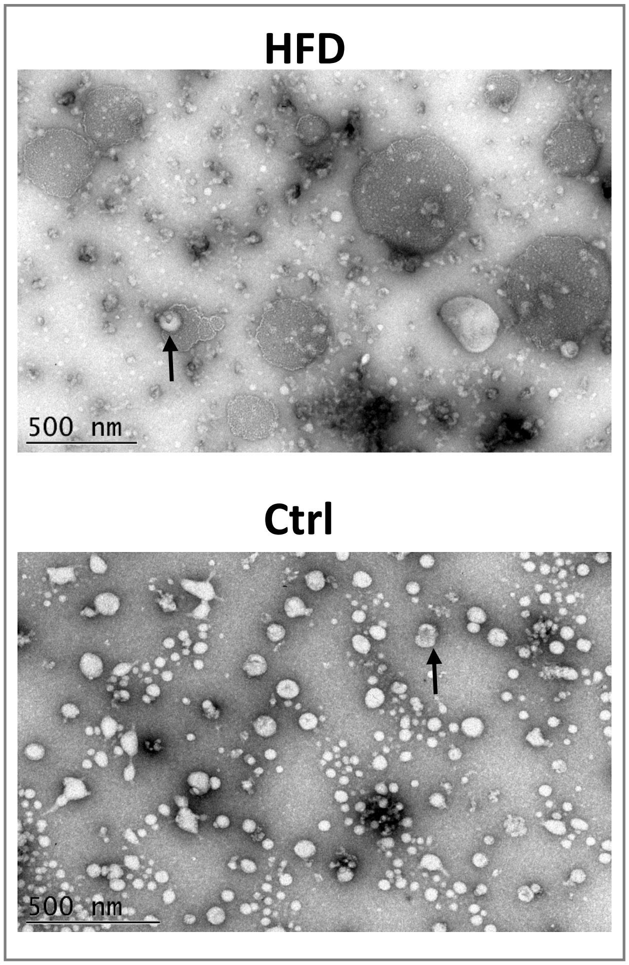

Micrographs of adEV: fixed in glutaraldehyde phosphate buffer, adsorbed on 0.3% formvar and stained in 2.5% uranyl acetate; black arrows indicate extracellular vesicles, TEM, JEOL JEM-1011 equipment; n=1 per group, HFD; high-fat diet, Ctrl; control.
