# Supplementary material for: The association between the size of adipocyte-derived extracellular vesicles and fasting serum triglyceride-glucose index as proxy measures of adipose tissue insulin resistance in a rat model of early-stage obesity
Source: Front Nutr. 2024 Jul 1;11:1387521. doi: 10.3389/fnut.2024.1387521 (PMC11247012; doi:10.3389/fnut.2024.1387521)
Supplement: Supplementary file 3 [file Table_1.pdf]

TABLE 1S. **Multivariate regression analysis predicting IR according to TAG/HDL-c.**

	Model SW		Model S1		Model S2		Model S3		Model S4	
**TAG/HDL-c log_{10}**	**B**	**p value**	**B**	**p value**	**B**	**p value**	**B**	**p value**	**B**	**p value**
adEV size (nm)	---	---	0.0010	0.166	0.0012	0.107	0.0014	0.074	0.0013	0.107
Zeta potential (mV)	---	---	---	---	---	---	---	---	---	---
Final weight gain (g)	---	---	0.0021	0.163	---	---	---	---	---	---
Body weight (g)	---	---			0.0012	0.399	---	---	---	---
EpAT/BW log_{10}	---	---	---	---	---	---	---	---	0.6759	0.395
Body Fat (%)	---	---	---	---	---	---	-0.001	0.889	---	---
FE (kcal/g)	19.19	0.034	---	---	---	---	---	---	---	---
Group (HFD)	---	---	---	---	---	---	---	---	-0.003	0.981

adEV: adipocyte-derived extracellular vesicles; EpAT/BW: epididymal adipose tissue/body weight; FE: Food efficiency rate; HFD: High-fat diet; TAG/HDL-c: triglyceride- high-density lipoprotein cholesterol. Model SW was obtained with a stepwise approach. Models S1 to S4 tested the sensitivity of model SW by adjusting it for each of the variables rejected by the stepwise approach. Values correspond to the beta coefficient.
